# Anticentromere antibody induced by immunization with centromere protein a and Freund’s complete adjuvant may interfere with mouse oocyte meiosis

**DOI:** 10.1186/s12958-021-00737-w

**Published:** 2021-04-01

**Authors:** Ying Ying, Shuang Liu, Yixuan Wu, Sichen Li, Qing Huang

**Affiliations:** Department of Obstetrics and Gynecology, Key Laboratory for Major Obstetric Diseases of Guangdong Province, Reproductive Medicine Center, The Third Affiliated Hospital of Guangzhou Medical University, 63 Duobao Road, Liwan District, Guangzhou, China

**Keywords:** Anticentromere antibody, Oocyte meiosis

## Abstract

**Background:**

Anticentromere antibody (ACA) is a member of the antinuclear antibody (ANA) family, and recent studies have found that ACA may be associated with oocyte maturation disorders; however, the possible mechanism behind this phenomenon remains unknown. We conducted this study to investigate whether ACA could penetrate into the living oocytes and interfere with oocyte meiosis in a mouse model.

**Methods:**

We divided mice into three groups: human recombinant centromere protein-A (human CENP-A, HA) and complete Freund’s adjuvant (CFA) were used to immunize mice for the study group (HA + CFA), and mice injected with CFA (CFA group) or saline (Saline group), respectively, served as controls. After immunization, serum anti-CENP-A antibody was detected by indirect immunofluorescence assay (IIFT) and enzyme-linked immunosorbent assay (ELISA). Chromosome alignment and intracellular IgG localization in MI- and MII-stage oocytes were investigated by immunofluorescence analysis.

**Results:**

Positive ACAs were successfully induced by immunization with CENP-A and CFA, and results showed that the serum level of anti-CENP-A antibody was significantly higher in the HA + CFA group compared with the control groups. There was marked increase of chromosome misalignments in MI and MII oocytes in the HA + CFA group compared to the control groups. However, no oocytes from any of the three groups showed intracellular antibody immunofluorescence.

**Conclusions:**

The development and maturation of oocytes were impaired in peripheral ACA positive mice, which exhibited severe chromosomal misalignments in metaphase meiosis; however, no evidence of ACAs entering the oocytes was observed, thus the underlying mechanism needs further exploration.

## Background

Evidence shows that different types of antinuclear antibody (ANA) can enter living cells [1–3]. In 1999, a study showed that early-stage mouse embryos cultured with purified IgG from ANA positive serum exhibited strong fluorescence and had significant growth impairment [4]. We also previously identified the presence of ANA in follicular fluid and early-stage embryos in women who were ANA-positive in peripheral blood, suggesting that ANA may penetrate into the embryo [5].

Recent retrospective clinical studies showed that disorders of oocyte maturation and early embryonic development were found in women with positive anticentromere antibody (ACA) in their peripheral blood [6, 7]. ACAs belong to the antinuclear antibody spectrum and are regarded as important serological markers for systemic sclerosis (SSc), particularly the form of SSc known as CREST (calcinosis cutis, Raynaud’s phenomenon, esophageal dysfunction, sclerodactyly and telangiectasia) syndrome [8–11]. More recently, we found that embryos cultured with anti-CENP-A (centromere protein-A) antibody had significant growth impairment and/or high mortality [12]. CENP-A is an important protein of the CENP system and one of the major antigens for ACA in patients with SSc [11, 13, 14]. The specific binding between ACA and CENPs is almost completely unrestricted among different species.

In the present study, we hypothesized that ACA might be the major antibody in the ANA family to have adverse impacts on oocyte and embryo potential. Recombinant human CENP-A and CFA were used to induce ACA production in mice, then mouse MI- and MII-stage oocytes were cultured to investigate chromosome alignment and intracellular IgG localization by immunofluorescence assay in order to explore the involvement of ACA in impaired oocyte meiosis.

## Methods

### Mice

Six-week-old wild-type female ICR mice were purchased from Guangdong Medical Laboratory Animal Center. All experiments and procedures were approved by the Ethics Committee of The Third Affiliated Hospital of Guangzhou Medical University (approval number 2017–156).

### Immunization protocols

Mice were immunized subcutaneously in the lower back with 150 μg CENP-A (Biorby Ltd., UK) emulsified in a total volume of 300 μl complete Freund’s adjuvant (CFA, Sigma-Aldrich, Germany) prepared using a high-speed tissue homogenizer (modified based on the literature [15]). After the initial immunization, the second and third immunization boosters were performed at an interval of 2 weeks. Mice subcutaneously injected with CFA or saline served as controls resulting in three groups: HA + CFA, CFA alone, and saline. Blood samples were collected by retroorbital bleeding or bleeding through the tail vein.

### Oocyte collection and culture

The mice were sacrificed by cervical dislocation and disinfected with 70% alcohol. Prepare a 35 mm petri dish with M2 medium (Sigma-Aldrich, USA) preheated to 37 °C. Open the abdominal cavity, find the ovaries at the upper end of the uterus, bluntly separate the ovaries and immerse in the medium. Ovarian tissue was chopped to make ovarian tissue homogenate, the GV-stage oocytes were sucked out under 40x magnification and cultured in Quinn’s Advantage TM Cleavage Medium (SAGE, USA).

After culture for 8 and 12 h, the time points when most oocytes had reached the MI and MII stages, respectively, the oocytes were harvested for immunofluorescence analysis.

### Serum anti-CENP-A detection

Mouse serum anti-CENP-A antibody was measured by two methods: indirect immunofluorescence test (IIFT) and enzyme-linked immunosorbent assay (ELISA). The IIFT kit for antinuclear IgG antibodies (Euroimmun AG, Germany) is the gold standard for the determination of antibodies against nuclear antigens. Since the sample to be tested was mouse serum, we substituted the anti-human secondary antibody in the original kit with the Alexa Fluor 488-conjugated goat anti-mouse IgG (Cell Signaling Technology, USA). The specific ELISA kit for assessing anti-CENP-A antibody was purchased from Wuhan Bolehui Biotechnology Co, LTD. The CENP-A (Biorby Ltd., UK) was used to coat the ELISA plates and the CENP-A antibody (Biorby Ltd., UK) was used as the standard. The experimental procedures were carried out according to the manufacturer’s instructions.

### Immunofluorescence staining for chromosome alignment and intracellular IgG localization in oocytes

After 8 or 12 h of culture, MI- or MII-stage oocytes were isolated for the immunofluorescence assay, to assess chromosome alignment or to detect the anti-CENP-A antibody signal in oocyte from serum-ACA-positive mice.

The procedures for the immunofluorescence assay were as follows: oocytes were fixed in 4% polyoxymethylene and permeated with 0.5% Triton X-100 (Sigma, USA), followed by sealing in 5% normal donkey serum (Jackson Immunoresearch, USA). For staining of Spindle microtubules, oocytes were incubated overnight at 4 °C with rat anti-tubulin antibody (Abcam, United Kingdom, 1:800 dilution). After three washes in washing buffer, oocytes were incubated with Alexa Fluor® 488 AffiniPure Donkey anti-Rat IgG (Jackson Immunoresearch, USA, 1:500 dilution) for 1 h at room temperature, rinsed, incubated with 1 μg/mL DAPI (Cell Signaling Technology, USA) for 15 min, rinsed again, and fixed in a dish for subsequent microscopic observation. For intra-oocyte IgG staining, following incubation with Alexa Fluor® 647 AffiniPure Donkey anti-Mouse IgG (Jackson Immunoresearch, USA, 1:500 dilution) for 2 h at room temperature, oocytes were washed 3 times in washing buffer, and co-stained with 1 μg/mL DAPI for 15 min. These oocytes were mounted on glass slides and examined with a confocal laser-scanning microscope (LSM780; Zeiss GmbH, Germany).

### Statistical analysis

was performed using SPSS 13 (SPSS, Inc., Chicago, IL, USA). All continuous variables were expressed as mean ± standard deviation (SD). The Mann-Whitney U test was used to evaluate differences among groups, and analysis of variance followed by Bonferroni adjustment was used for multiple comparisons. *P* < 0.05 was considered statistically significant.

## Results

### Mice immunized with CENP-A developed marked anti-CENP-A responses

**Fig. 1:**
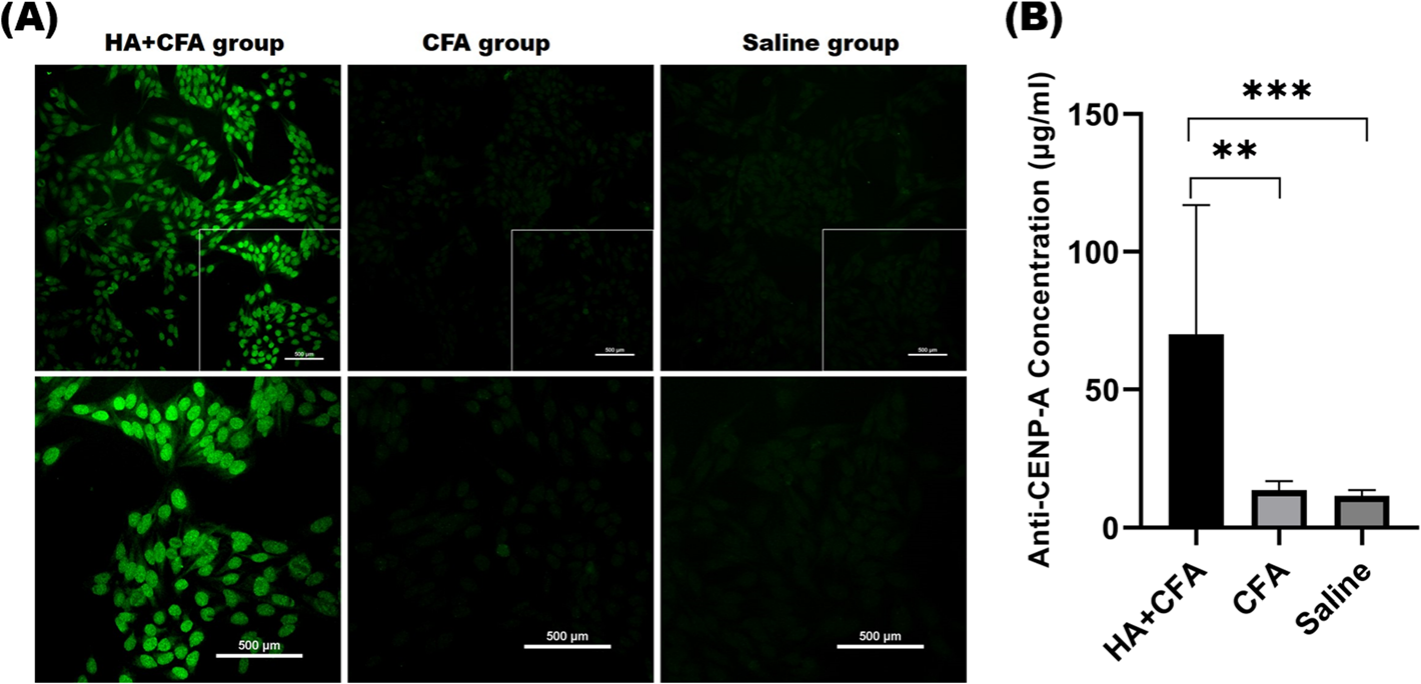
Determination of anti-CENP-A antibody in mouse serum after treatment. **a** IIFT test for ANA-IgG to determine anti-CENP-A in mouse serum. Positive ANA was detected in serum samples from mice in the HA + CFA group, while none of the serum samples from the CFA and saline groups showed a fluorescence signal (*n* = 10 for each group, with an original magnification × 200). **b** Serum levels of anti-CENP-A antibody measured by the ELISA. Mice treated with CENP A and CFA exhibited significantly higher levels of anti-CENP-A antibody compared to mice treated with saline or CFA alone. Each histogram represents the mean ± SD obtained from 10 mice in each group, **p < 0.01 vs. CFA-treated mice, ***p < 0.001 vs. saline-treated mice

After immunization with CENP-A emulsified in CFA, mouse serum anti-CENP-A antibody was measured by IIFT for ANA-IgG and ELISA. All serum samples from mice immunized with CENP-A were ANA positive compared to controlled mice (Fig. 1a). Mice immunized with CENP-A developed significantly higher levels of anti-CENP-A antibody (70.15 ± 46.78 μg/ml) compared to mice treated with CFA (13.74 ± 3.23 μg/ml) or saline (11.54 ± 2.11 μg/ml). (Fig. 1b).

### No IgG immunofluorescence was detected in oocytes from serum-ACA-positive mice, while these oocytes showed abnormal chromosome alignment

**Fig. 2:**
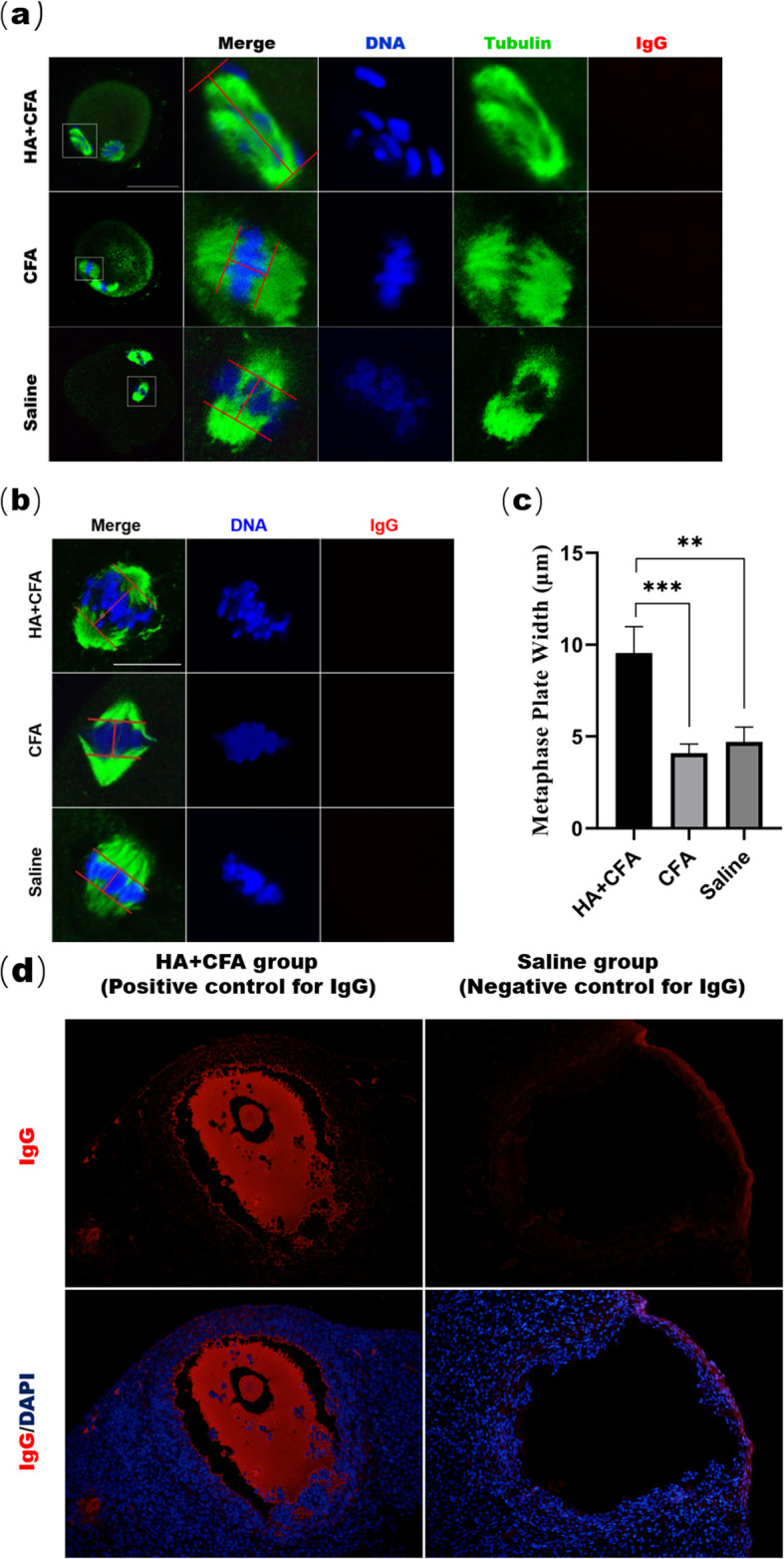
Misaligned chromosomes in MI- and MII-stage oocytes from serum-ACA-positive mice. In the HA + CFA group, most MI- (**a**) and MII-stage (**b**) oocytes showed severely misaligned chromosomes, while in the control groups, most oocytes showed normal chromosome alignment. None of the oocytes from the three groups exhibited IgG fluorescence. DNA (blue), tubulin (green), IgG (red). Scale bars: 10 μm. **c** Metaphase plate width was determined by measuring the axis distance between the two lines at the edges of the DNA. Data are expressed as mean ± SD of ten MI-stage oocytes in each group. **P < 0.01; ***P < 0.001. **d** Positive and negative control for IgG stain. Mouse ovarian tissue sections from HA + CFA and Saline groups were stained with the anti-mouse IgG. The red fluorescence of IgG, predominantly distributed in the follicular fluid, was observed in mice from the HA/CFA group, and no fluorescence of IgG was visualized in mice from the Saline group, which were served as positive and negative controls for IgG stain, respectively (with an original magnification × 200)

We hypothesized that ACA enters the oocyte and causes chromosome misalignment during oocyte meiosis in serum ACA positive mice. To test the hypothesis, MI- and MII-stage oocytes were cultured and the results showed that MII-stage oocytes from ACA-positive mice contained misarranged chromosomes, but no intracellular IgG signal was observed in oocytes from serum-ACA-positive mice (Fig. 2a). The incidence of chromosome misalignments in MII-stage oocytes was significantly increased in the HA + CFA group (45.5%; 10 of the 22 oocytes contained abnormally arranged chromosomes) compared to CFA group (18.52% [5/27]) and Saline group (15.0% [3/20]).

Similarly, immunofluorescence showed that in MI-stage oocytes from the HA + CFA group, various chromosomes were incapable of aligning at the middle plate, and none of the oocytes from each group exhibited an intracellular IgG signal (Fig. 2b and c).

## Discussion

Several clinical studies have reported that the presence of ACA can exert adverse effects on oocyte maturation and early-stage embryo development [6, 7], and our research has focused on identifying the underlying mechanisms behind this. As early as 1990, researchers found that microinjection of serum containing ACA into mouse oocytes could hinder chromosome aggregation and cause meiotic arrest in interphase, or mitotic arrest in prometaphase [16]. Recently, we found that embryos cultured with anti-CENP-A antibody exhibited immunofluorescence on the nucleus and had significant growth impairment [12]. Thus, we proposed a hypothesis that oocytes could be a direct target for ACA, and that ultimately ACA interferes with meiosis by entering the oocyte directly.

The results of the present study showed significantly increased incidence of chromosome misalignments in MI- and MII-stage oocytes in mice with positive ACA in their peripheral blood, which was induced by treatment with CENP-A. However, no intracellular antibody fluorescence was observed in these meiosis-disturbed oocytes, which suggests that the effects of ACA on oocyte maturation might not be achieved through direct access into the oocyte.

Chromosome segregation is notoriously error-prone in female meiosis, and most aneuploidies appear to be caused by lack of faithful segregation of chromosomes during meiosis. The stability of the oocyte genome depends on accurate segregation of chromosomes, which requires dynamic interaction between microtubules and kinetochores. The present study showed that chromosomes were not able to congregate along the equatorial plate during metaphase, which suggests that the interaction between kinetochores and microtubules may have been disrupted. As we know, kinetochores provide the major microtubule attachment sites on chromosomes; therefore, faithful attachment between microtubules and chromosomes depends on the kinetochore [17, 18]. As such, we hypothesized that the presence of ACA may interfere with the attachment between kinetochores and microtubules. We were however unable to elucidate the underlying mechanism in the present study; therefore, further exploration of the mechanisms underlying the involvement of ACA in impaired oocyte development is required.

Future research will aim to verify the observed interference of oocyte meiosis in ACA positive women through clinical studies and to further explore the relationship between ACA and impaired oocyte meiosis and underlying pathophysiological mechanisms.

## Conclusions

The presence of ACA induced by immunization with CENP-A may impair oocyte meiosis. The underlying interference mechanism requires further exploration.

## Data Availability

The datasets used and/or analyzed in the current study are available from the corresponding author upon reasonable request.

## Abbreviations

ANA: Antinuclear antibody
ACA: Anticentromere antibody
CENP: Centromere protein
CFA: Complete Freund’s adjuvant
CREST: Calcinosis cutis, Raynaud’s phenomenon, esophageal dysfunction, sclerodactyly and telangiectasia
IIFT: Indirect immunofluorescence test
